# The Effects of Swiprosin-1 on the Formation of Pseudopodia-Like Structures and β-Adrenoceptor Coupling in Cultured Adult Rat Ventricular Cardiomyocytes

**DOI:** 10.1371/journal.pone.0167655

**Published:** 2016-12-16

**Authors:** Franziska Nippert, Rolf Schreckenberg, Antonia Hess, Martin Weber, Klaus-Dieter Schlüter

**Affiliations:** Institute of Physiology, Justus-Liebig-University, Giessen, Germany; Cincinnati Children's Hospital Medical Center, UNITED STATES

## Abstract

**Background:**

Recent findings suggest that adult terminally differentiated cardiomyocytes adapt to stress by cellular de- and redifferentiation. In the present study we tested the hypothesis that swiprosin-1 is a key player in this process. Furthermore, the relationship between swiprosin-1 and β-adrenoceptor coupling was analyzed.

**Methods:**

In order to study the function of swiprosin-1 in adult rat ventricular cardiomyocytes (ARVC) they were isolated and cultured in a medium containing 20% fetal calf serum (FCS). Changes in cell morphology of ARVC during cultivation were quantified by light and confocal laser scan microscopy. Small interfering RNA (siRNA) was used to reduce the expression of swiprosin-1. The impact of calcium on swiprosin-1 dependent processes was investigated with Bapta-AM. Immunoblot techniques and qRT-PCR were performed to measure mRNA and protein expression.

**Results:**

In culture, ARVC first lost their contractile elements, which was followed by a formation of pseudopodia-like structures (spreading). Swiprosin-1 was detected in ARVC at all time points. However, swiprosin-1 expression was increased when ARVC started to spread. Reduction of swiprosin-1 expression with siRNA delayed ARVC spreading. Similarly, Bapta-AM attenuated swiprosin-1 expression and spreading of ARVC. Furthermore, swiprosin-1 expression correlated with the expression of G protein-coupled receptor kinase 2 (GRK2). Moreover, silencing of swiprosin-1 was associated with a down regulation of GRK2 and caused a sensitization of β-adrenergic receptors.

**Conclusion:**

Swiprosin-1 is required for ARVC to adapt to culture conditions. Additionally, it seems to be involved in the desensitization of β-adrenergic receptors. Assuming that ARVC adapt to cardiac stress in a similar way, swiprosin-1 may play a key role in cardiac remodeling.

## Introduction

Neonatal cardiomyocytes have the ability to perform mitosis, however this capability vanishes within the first week post-partum. Terminally differentiated adult cardiomyocytes have lost the ability to proliferate [1]. However, cardiomyocytes are able to adapt to cardiac stress like hypertension, cell loss and aging. Recent findings suggest that adaptation is a complex process of cellular dedifferentiation and redifferentiation [2–6]. Adult rat ventricular cardiomyocytes (ARVC) in culture perform severe structural changes including sarcomere disassembly and reformation [7]. This is accompanied by a reexpression of fetal-type genes like β-myosin heavy chain (β-MHC) and α-smooth muscle actin (α-sm-actin) [8,9]. In culture ARVC form new sarcomeres alongside actin-driven stress fibers. This is preceded by the formation of pseudopodia-like structures, a process known as cell spreading. As a result, ARVC in culture transform into widespread, polymorphic cells [9]. The trigger that induces spreading is still unknown. We hypothesize that swiprosin-1, an actin-binding protein, plays a key role in this process. In a dimeric form Swiprosin-1, also known as EF-Hand Domain Family Member D2 (EFhd2), stabilizes F-actin filaments by blocking the binding site of cofilin. Cofilin is needed for the depolymerization of F-actin [10]. To date, swiprosin-1 has been only described in immune cells and in non-lymphatic brain tissue [10–12]. In immune cells it triggers the formation of lamellopodia which enable macrophages to migrate [10–12]. With the present study, we hypothesize that swiprosin-1 is required for the formation of pseudopodia-like structures (spreading) in ARVC.

The heart responds to pathological stress like hypertension or ischemia by hypertrophy, which eventually leads to maladaptive cardiac remodeling and finally heart failure. Some of these maladaptive processes are calcium-calcineurin-dependent [13–16]. However, not all changes linked to maladaptation may be explained by calcineurin activation, even though high diastolic calcium levels seem to be a trigger [13,15]. Notably, calcium is also required for swiprosin-1 activation by being involved in the formation of swiprosin-1 dimers which block the binding of cofilin [10,11]. Therefore, it may hamper cofilin activity. Activation of swiprosin-1 by calcium and its ability to stabilize actin stress fibers encouraged us to analyze whether ARVC express swiprosin-1, and whether swiprosin-1 is required for the formation of pseudopodia-like structures in these cells. The latter are necessary for the subsequent rearrangement of sarcomeres. Accordingly, we re-established the above described model of cultivation of ARVC. As a control molecule that has already been identified to be required in the process of spreading, oncostatin M was investigated [4]. Additionally, former studies have shown a reduction of β-adrenoceptor responsiveness under the same culture conditions that induce spreading of cardiomyocytes [17–19]. Therefore, we correlated swiprosin-1 expression with genes known to interfere with β-adrenoceptor-coupling.

Taken together, our study was done on the basis of recent discoveries that cardiac de- and redifferentiation as it occurs under culture conditions mimics features seen *in vivo* during cardiac remodeling [2,4]. We want to identify if swiprosin-1 plays a key role in the process of de- and redifferentiation and by that may be involved in the process of cardiac remodeling.

## Materials, Animals and Protocols

The investigation was conducted according to the Guide for the Care and Use of Laboratory Animals published by the US National Institute of Health (NIH Publication No. 85–23, revised 1996). The protocol was approved by the Justus Liebig University Giessen (permission number: 507_M). Sacrifice was performed under isoflurane anesthesia, all efforts were made to minimize suffering of the animals.

### Isolation and cultivation of adult rat ventricular cardiomyocytes

Ventricular cardiomyocytes of four month old male Wistar rats were isolated as described previously [20]. Briefly, the rat was sacrificed by cervical dislocation under deep anesthesia with isoflurane. The heart was immediately transferred into ice-cold saline solution. It was fixated on the cannula of a Langendorff perfusion system, followed by a 25 minute perfusion with Powell medium (80ml) containing collagenase (25mg) and CaCl_2_ (25μM) at 37°C. Subsequently, ventricular tissue was minced and incubated in 5ml of the re-circulated buffer for five minutes. The remaining cell solution was filtered through a nylon mesh (200μm). After centrifugation and stepwise addition of CaCl_2_ (200μM, 400μM and 1000μM) cells were plated on petry dishes, coated with 4% (vol/vol) fetal calf serum (FCS). After one hour, the medium (medium 199) supplemented with carnitine (2mM), creatin (5mM), taurine (5mM), penicillin-streptomycin (2%) and 20% FCS was refreshed. Cytosine-β-arabinofuranoside (10mM) was supplemented to hamper the proliferation of any contaminating cells. The incubator was held at 37°C.

Small interfering RNA (siRNA; FlexiTube siRNA Swiprosin, QIAGEN, Netherlands) at a concentration of 0.05μM was used to decease the expression of swiprosin-1. Scrambled siRNA (“scRNA”, All Stars Negative Control siRNA, QIAGEN, Netherlands) served as negative control and was applied in the same concentration. This RNA interference took place once after washing the freshly plated ARVC.

Intracellular calcium was diminished by Bapta-AM (Molecular Probes ®invitrogen dectection technologies, USA). It was dissolved in DMSO (5μg/ml; Roth, Germany) and added to the plates in a concentration of 10μg/ml after the last washing step. Pure DMSO in a concentration of 5μg/ml was used as a control.

With each cell preparation 150 to 300 cardiomyocytes were evaluated per day and group by light microscopy. All counted cardiomyocytes were subdivided into four groups according to their appearance: “rod-shaped”, “round down”, “spreading” and “unusual appearance” (S1 Fig). The category “spreading” included all cardiomyocytes with pseudopodia-like structures. “Unusual appearance” included all ARVC with an irregular surface and no detectable intact cell membrane.

After five days in culture ARVC showed no expression of fibroblast markers (collagen-3), but of cardiac specific markers like ANP compared to isolated cardiac fibroblast after 24 hours in culture, which showed vice-versa results. Both, cardiac fibroblasts and ARVC expressed swiprosin-1 (Efhd2) on mRNA level (S2 Fig).

### Immunofluorescence staining of cardiomyocytes

In order to analyze the morphological and structural conversion of cardiomyocytes in culture confocal laser scan microscopy was performed. Phalloidin TRITC (Santa Cruz Biotechnology, Germany) was used to stain F-actin according to the manufacturers protocol. The day of cell isolation, labeled as day zero, was used as control. Briefly, cells were fixated with paraformaldehyde (4%). After permeabilization with TritonX-100 (0.2%) cardiomyocytes were incubated with Phalloidin-TRITC (10μMol). F-actin filaments appeared red in immunofluorescence microscopy.

### Immunoblot technique

Isolated ARVC were incubated with lysis buffer as described previously [21]. SDS page gel electrophoresis was conducted with the system of NuPAGE, Novex® (Life Technologies, USA). The expression of swiprosin-1 was detected by using a swiprosin-1 antibody produced in goat (Biorbyt Ltd., USA). A GRK-2 antibody produced in rabbit (Sigma-Aldrich, Germany) was used to investigate GRK-2 expression in ARVC. All measurements were normalized to the expression of GAPDH using an antibody produced in mice (Calbiochem®, Germany). Protein expression was quantified with horseradish peroxidase and a chemiluminescence machine from Peqlab.

### RT-PCR

Real-time quantitative RT-PCR in ARVC was performed as described before [22]. Total RNA was isolated using peqGOLD TriFast (Peqlab, Biotechnologie GmbH, Germany) according to the manufacturer`s protocol. After conversion of RNA into complementary DNA (cDNA) with reverse transcriptase, PCR was performed with iQ™SYBR® Green Supermix (BIO Rad, Germany). In order to detect unspecific binding melting curve analysis or DNA gels were performed. For experiments with quantitative real time RT-PCR, ARVC of four petri dishes (2ml) were combined in one sample. Each group contained twelve to fourteen samples. For correlation analyses all samples were analyzed. The primers used are listed in supplementary materials (S1 Table).

### Load free cell shortening

Determination of ARVC contraction was performed as described before [23,24]. ARVC were stimulated with two AgCl electrodes at 2Hz. Four measurements per cell were conducted and the mean of these measurements was used to define cell shortening. Isoprenaline (SERVA Feinbiochemica, Germany), a β-adrenoceptor agonist, was added five minutes before measurement in a concentration of 10μM. Additionally, ARVC were incubated for 24 hours with scRNA or siRNA directed against swiprosin-1 in a medium without FCS. All experiments were normalized to the control group of ARVC without any treatment.

### Statistics

Statistical analysis was performed using two-way ANOVA followed by Student-Newman-Keuls post hoc analysis or one-side ANOVA with Tukey test. If required, Student´s T-Test was performed with Levene Test to test for normal distribution of samples within a group. Man-Whitney-U post hoc analysis was conducted for samples without normal distribution. For the statistical calculation of all data SPSS 22.0 was used. A value of p < 0.05 was considered to be significant. Results are presented as mean ± standard deviation (SD) or mean ± standard error of the mean (SEM), indicated in the legend to the figures.

## Results

### Adult cardiomyocytes in culture

**Fig 1:**
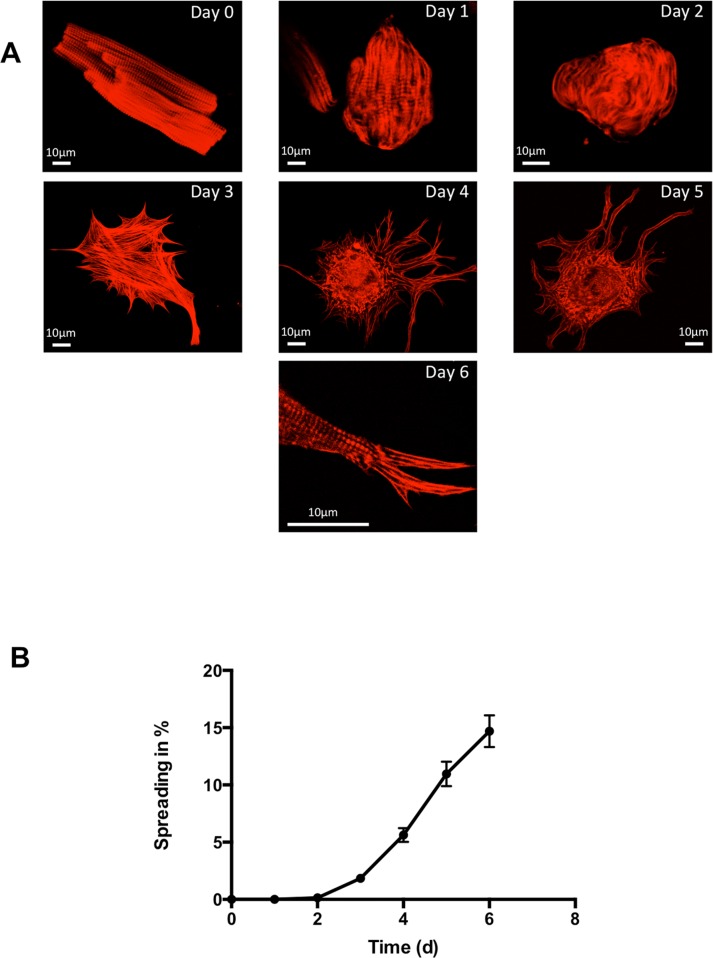
De- and redifferentiation of ARVC in culture with 20% FCS (A) Freshly isolated ARVC with their typical rod shape form (Day 0) completely rounded down by degrading sarcomeres starting in the periphery (Day 1) in the first days of culture. They lost all their contractile elements (Day 2) followed by formation of pseudopodia-like structures (spreading; Day 3–5) and subsequent reformation of their contractile elements indicating *de novo* sarcomerogenesis (Day 6). At day six in culture, cross striation was clearly detectable again. (B) Increase in cardiomyocytes with pseudopodia-like structures normalized to all counted cardiomyocytes (spreading in %) during cultivation time (n = 33 cell preparations). Data are presented as means ± SEM

**Fig 2:**
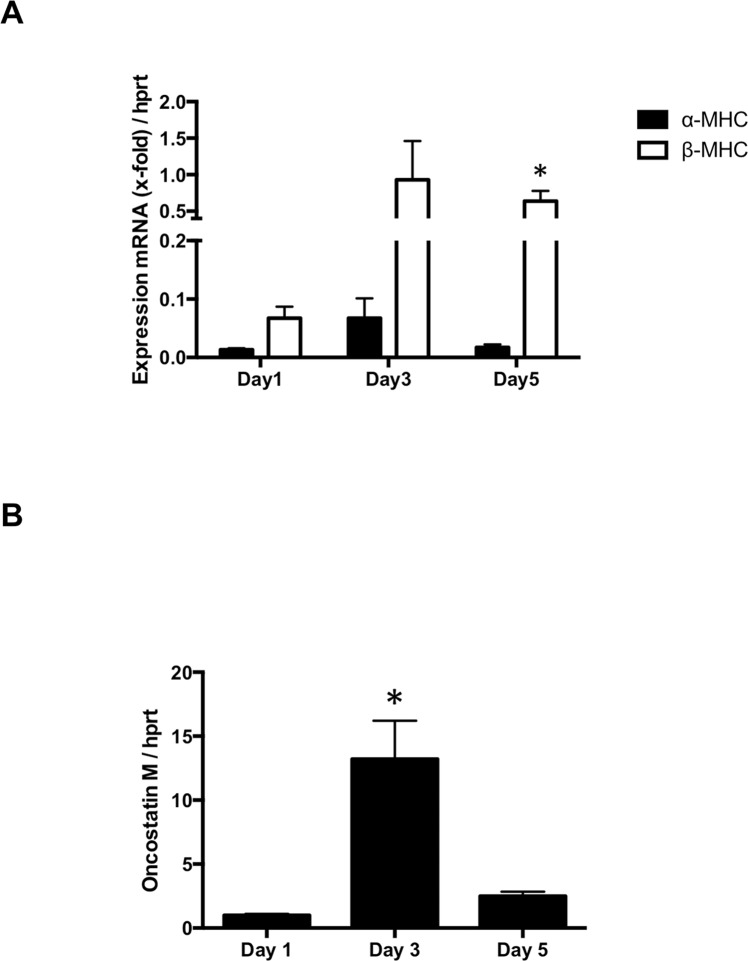
Expression of specific genes during cultivation (A) The expression of neonatal β-MHC mRNA increased whereas the expression of adult α-MHC stayed low at day one, three and five in culture (n = 12 samples; Man-Whitney-U-test). (B) The expression of oncostatin M increased during the first days in culture and decreased again at day five (n = 13 samples; one-side ANOVA with Tukey post hoc analysis). Data are presented as means ± SEM; *P ≤ 0.05

Changes in the shape of ARVC adapting to the culture conditions were investigated daily over a time period of six days. Freshly isolated ARVC were typically rod shaped with a clearly visible cross striation (Fig 1A Day 0). Changes in cell morphology were observed during the following days in culture. First ARVC lost all their contractile elements beginning in the periphery (Fig 1A Day 1 and Day 2). This was followed by a reformation, implicating *de novo* sarcomerogenesis. This reformation was preceded by the typical formation of pseudopodia-like structures (spreading, Fig 1A Day 3 –Day 6). *De novo* sarcomerogenesis started with the appearance of actin stress fibers (Fig 1A Day 3). Actin bundles initially appeared in the perinuclear region and formed newly assembled sarcomeres (Fig 1A Day 4 and Day 5). Latter grew along the preformed actin stress fibers into the periphery (Fig 1A Day 6). At the end of the cultivation period (Day 6), a typical cross striation from newly assembled sarcomeres in the spread ARVC was observed. Fig 1B displays the kinetic of the spreading process during cultivation. The fraction of ARVC showing pseudopodia-like structures at each time of examination are given as spreading in % (Fig 1B). Spreading started around day three and increased constantly during cultivation time, until 14.7% ± 1.39% ARVC showed pseudopodia-like structures at day six. This process was accompanied by reexpression of β-MHC, whereas the expression of α-MHC decreased during cultivation time (Fig 2A). Furthermore, oncostatin M was significantly increased, when the formation of pseudopodia-like structures started at day three (Fig 2B).

### Expression of swiprosin-1 during cultivation

**Fig 3:**
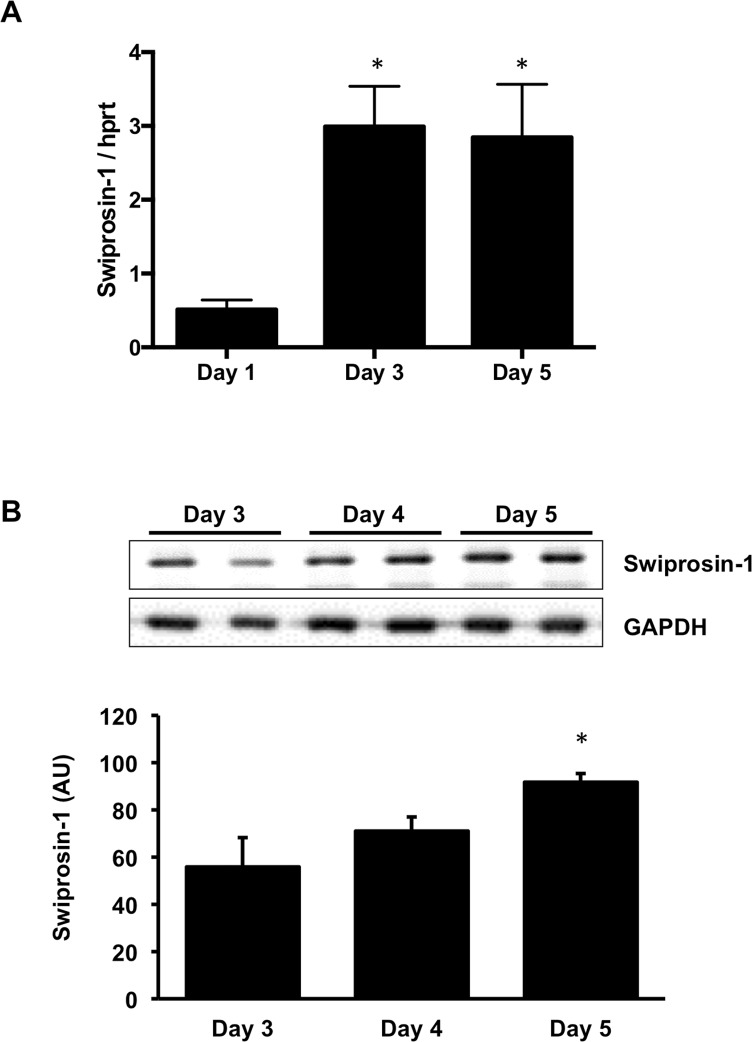
Expression of swiprosin-1 in ARVC (A) Expression of swiprosin-1 mRNA increased early during culture (n = 11–13 samples). (B) On protein level expression of swiprosin-1 decreased in the first days of culture (data not shown), followed by a significant increase in the last days of culture (n = 4 cell preparations). Data are presented as means ± SD; *P ≤ 0.05; one-side ANOVA with Tukey post hoc analysis, AU = arbitrary units

To our knowledge we are the first to provide evidence that swiprosin-1 is expressed in ARVC. Changes in the expression of swiprosin-1 in ARVC during cultivation are shown in Fig 3A and 3B. Swiprosin-1 mRNA levels increased during cultivation (day 1: 0.51-fold ± 0.13; day 3: 2.99-fold ± 0.55; day 5: 2.85-fold ± 0.72 compared to day 0) (Fig 3A). Protein expression of swiprosin-1 decreased throughout the first three days (data not shown) followed by a significant increase when pseudopodia-like structures appeared (Fig 3B). Typically, mRNA expression of swiprosin-1 increased before protein expression. Thus, cell spreading is associated with increased swiprosin-1 expression during cultivation.

### Swiprosin-1 is needed for *de novo* sarcomerogenesis in cultured ARVC

**Fig 4:**
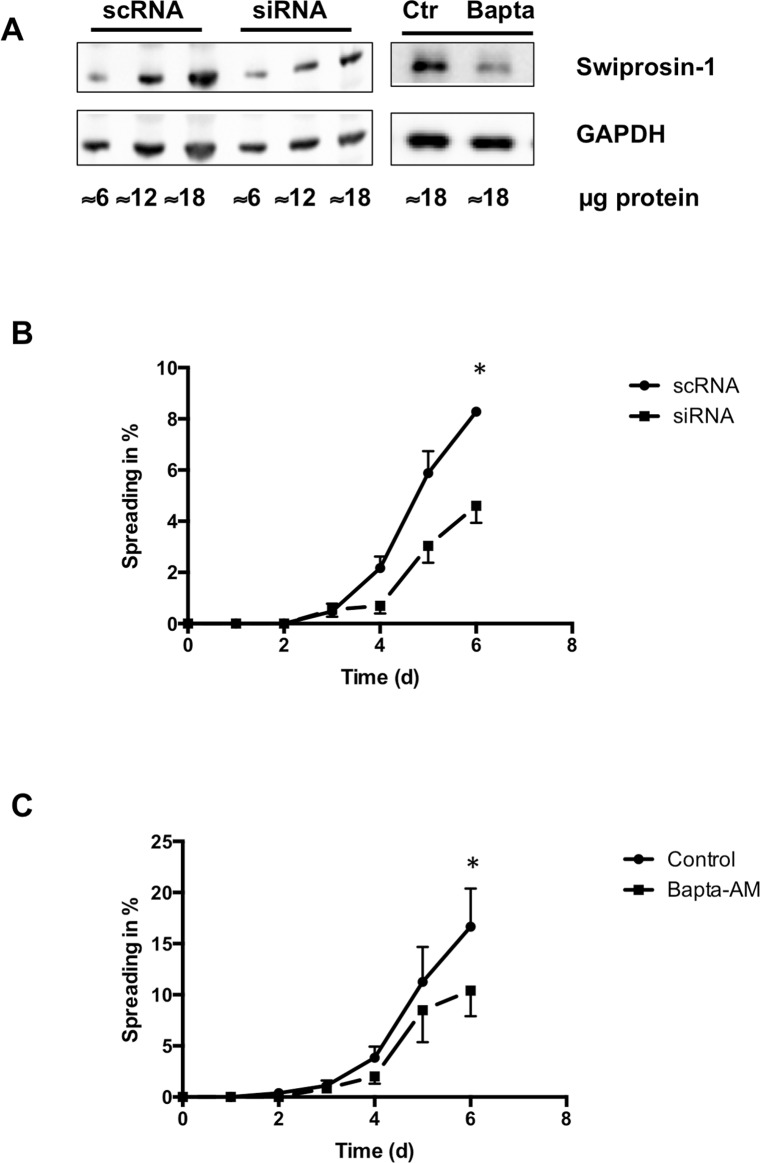
Blocking the translation of swiprosin-1 with siRNA and decreasing the intracellular calcium with Bapta-AM attenuated spreading in ARVC. (A) siRNA against swiprosin-1 (0.05μM) caused a down regulation in protein expression for swiprosin-1 compared to scRNA at day 2 in culture. Additionally, Bapta-AM (10μg/ml) decreased the expression of swiprosin-1 on protein level, too. (B) Through the whole cultivation a constant down regulation of spreading in ARVC treated with siRNA against swiprosin-1 was detectable. (C) Bapta-AM led to a constant decrease in spreading, too. Data are presented as means ± SEM; n = 3–4 cell preparations (n = 800–1000 cells); *P ≤ 0.05; unpaired T-Test or Man-Whitney-U-Test, Ctr = control group, Bapta = ARVC treated with Bapta-AM

In order to investigate the role of swiprosin-1 in the spreading process more closely, experiments with siRNA directed against swiprosin-1 were performed. Immunoblot analysis confirmed a significant reduction to 74.77% ± 11.88% of swiprosin-1 at protein level with siRNA directed against swiprosin-1 compared to scramble RNA (scRNA) (Fig 4A). Addition of siRNA directed against swiprosin-1 delayed spreading of ARVC. At day six, siRNA against swiprosin-1 reduced spreading by 55.61% ± 14.34% compared to ARVC treated with scRNA (Fig 4B). Additionally, fewer cells performed spreading in the presence of siRNA against swiprosin-1 at day four (p = 0.052) and day five (p = 0.059). Although the number of ARVC performing cell spreading was lower in cultures treated with siRNA, the ARVC that still showed pseudopodia-like structures, performed the same dedifferentiation and redifferentiation process as seen in control conditions. First they completely lost their contractile apparatus followed by a reformation of actin-stress fibers and *de novo* sarcomerogenesis (S3 Fig).

### Effect of intracellular calcium modulation on cell spreading

Calcium is required to activate swiprosin-1 [10,11]. We hypothesize that an alteration in the intracellular calcium levels may influence the ability of adult cardiomyocytes to spread. Bapta-AM, a known cell-permeable calcium chelator, caused a constant decrease in spreading of ARVC compared to the control group. At day six, Bapta-AM decreased spreading by 62.42% ± 24.10% compared to control (Fig 4C). On protein level a significant down regulation (49.89% ± 9.87%) of swiprosin-1 was found in Bapta-AM treated cardiomyocytes compared to control (Fig 4A).

### Swiprosin-1 expression correlates with GRK2 expression and causes β-adrenoceptor desensitization

**Fig 5:**
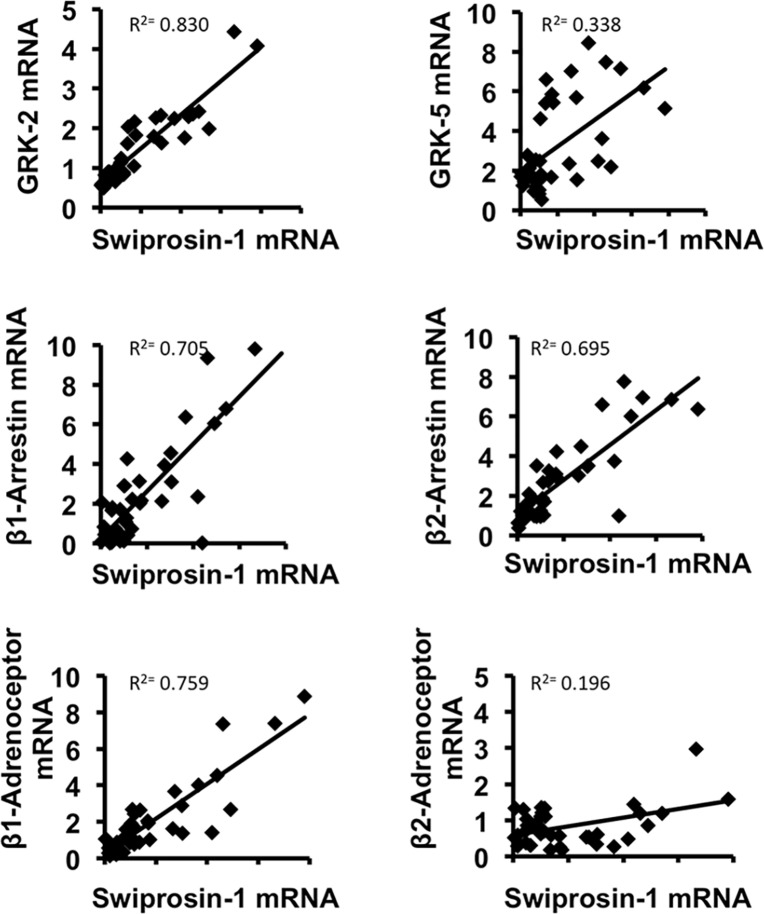
Linear correlation between swiprosin-1 and β_1_- and β_2_-adrenoceptor as well as with proteins known to interfere with β-adrenoceptors (n = 44 samples). ARVC were harvested at day 1,3 and 5 in culture. Changes in swiprosin-1 expression were correlated with genes of interest.

**Fig 6:**
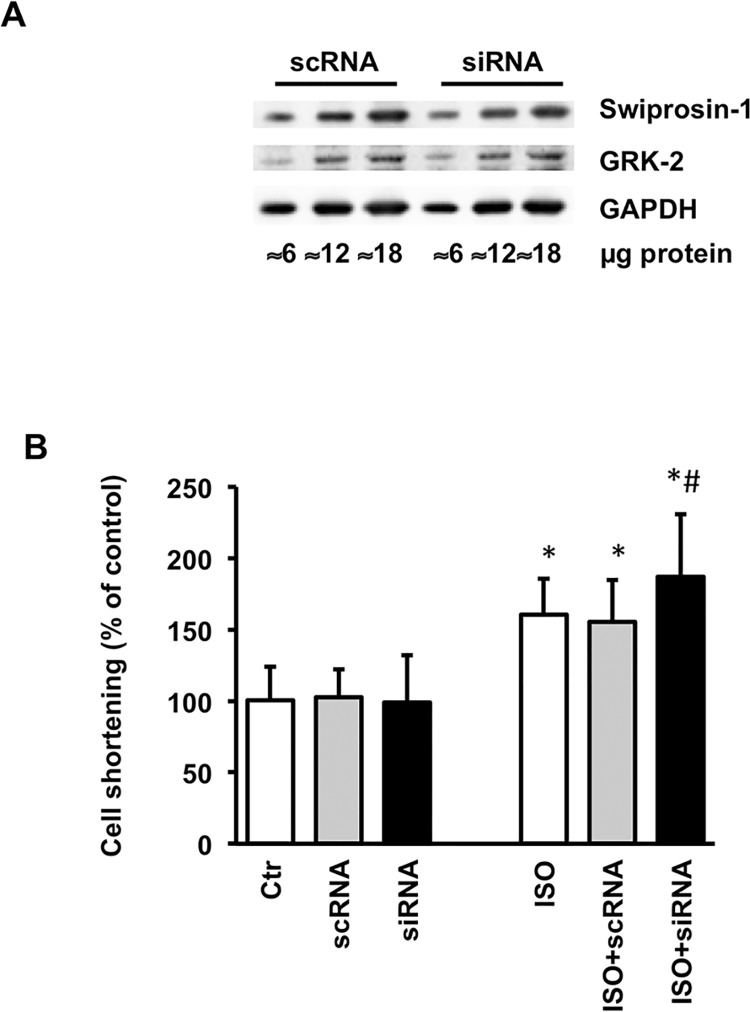
Swiprosin-1 caused β-adrenoceptor desensitization by interaction with GRK2. (A) GRK2 protein level was reduced in ARVC treated with siRNA against swiprosin-1 (0.05μM) compared to scRNA at day 1 in culture. (B) In cell shortening of ARVC after 24h in culture between control, scRNA and siRNA for swiprosin-1 no difference was detectable. Adding isoprenaline (ISO; 10μM) to control ARVC and ARVC treated with scRNA led to an increase in cell shortening ability. This effect was increased in ARVC treated with siRNA, thus down regulation of swiprosin-1 led to down regulation of GRK2 and therefore to an increased responsiveness to the β-adrenoceptor agonist isoprenaline. Data are means ± SD; n = 72 cells; *P ≤ 0.05 vs. control: #p ≤ 0.05 vs. ISO; two-side ANOVA and post-hoc test (Student-Newman-Keuls)

Experiments with ARVC in culture showed a change in the responsiveness of β_1_- and β_2_- adrenoceptors [17–19]. As swiprosin-1 significantly affected the adaptation to culture conditions during which also changes in β-adrenoceptor coupling were observed, we correlated the expression of swiprosin-1 *in vitro* with genes known to interfere with β-adrenoceptors. The following four proteins showed a distinct positive correlation with the expression of swiprosin-1: G protein-coupled receptor kinase 2 and 5 (GRK2 and GRK5), β1-arrestin and β2-arrestin (Fig 5). All of these proteins are proved to be involved in the desensitization of β-adrenoceptors. Additionally, a positive correlation between the expression of swiprosin-1 and β_1_- and β_2_-adrenoceptors was detected. The highest correlation with swiprosin-1 was found for GRK2 (R^2^ = 0.830). Hence, swiprosin-1 may cause β-adrenoceptor desensitization via co-regulation of GRK2. To investigate this assumption, the isoprenaline responsiveness of ARVC during electric stimulation was tested and immunoblot analyses of ARVC treated with siRNA against swiprosin-1 were performed. Western Blots showed a decrease in protein level of GRK2 in ARVC treated with siRNA against swiprosin-1 compared to ARVC treated with scRNA (Fig 6A). Load free cell shortening (2Hz) showed no difference in basal contractile responsiveness between ARVC treated with scRNA, siRNA or vehicle (Fig 6B). However, controls increased their contractile amplitude as a response to β-adrenergic stimulation with isoprenaline. Interestingly, this contractile responsiveness was further increased in ARVCs treated with siRNA directed against swiprosin-1 (Fig 6B). The same effect could be detected with siRNA against GRK2 (Rolf Schreckenberg, personal communication). Summarized, the data suggest a functional relevant GRK2 down regulation in swiprosin-1 depleted ARVC.

## Discussion

This study reports three new findings. First, ARVC constitutively express swiprosin-1 as shown here in ARVC on mRNA and protein level. Second, swiprosin-1 is required to initiate the formation of pseudopodia-like structures (spreading), a process necessary to adapt to culture conditions. Third, the expression level of swiprosin-1 is associated to that of GRK2 and thereby to β-adrenoceptor responsiveness.

In the present study we successfully re-established the cultivation model of adult cardiomyocytes, which was described in the 1980s [8,9,25–27]. ARVC performed a severe remodeling during cultivation. First the cells lost their contractile elements accompanied by a transition from a rod-shaped morphology to a round form. Subsequently, ARVC formed pseudopodia-like structures (spreading) and finally emerged as polymorphic cells with an intracellular net of actin stress-fibers. Along these stress fibers new sarcomeres were formed. After six days in culture, the typical cross striation of sarcomeres reappeared in ARVC. This change was accompanied by replacing α-MHC by β-MHC. As expected, expression of oncostatin M, known to trigger the process of de- and redifferentiation was increased during cultivation [4]. Thus, within six days the process of de- and redifferentiation of ARVC, which is a well-known phenomenon, could be observed in our study. In this process ARVC showed a mixture between the fetal and embryonic cell-like cell type, but remained highly differentiated [25]. For example, they formed new sarcomeres alongside actin stress fibers like adult terminal differentiated cardiomyocytes do, whereas fetal cardiomyocytes start sarcomerogenesis at the sarcolemma [28]. The process of de- and redifferentiation enables ARVC to adapt to new environments and circumstances [2–6].

We showed with this study, swiprosin-1 is constitutively expressed in ARVC on mRNA and protein level. By hampering the translation of swiprosin-1 with siRNA, a significant reduction in spreading was detected in ARVC. Consequently, swiprosin-1 seems to be required for the adaptation of ARVC to culture conditions. According to the manufacturers’ protocol, the intracellular level of siRNA is sufficient to maintain the gene silencing effect for five to six days. Although, this cannot be certain for the last days in culture an effect of siRNA directed against swiprosin-1 was detected on protein level in the first days of cultivation. The main effect of siRNA directed against swiprosin-1 is a delay in the induction of the spreading process. The ARVC treated with siRNA which showed spreading, performed a similar process of dedifferentiation and redifferentiation as seen in control conditions. According to the treatment protocol it is possible, that either not all the swiprosin-1 in the cardiomyocytes was blocked or that not all cardiomyocytes absorbed the small interfering RNA. Because we could only detect a 25% decrease of swiprosin-1 on protein level and no transfection medium was used to protect the sensitive cardiomyocytes, we suspect that ARVC exhibiting the typical spreading behavior as seen under control conditions did not absorb the siRNA against swiprosin-1. Former researchers established that swiprosin-1 is required for the formation of lamellopodia in macrophages and by that for the formation of contractile elements enabling immune cells to migrate [10–12]. Taking our present results into consideration we proved that swiprosin-1 plays a similar role in cardiomyocytes, in which the formation of pseudopodia-like structures is required for adaptation to culture conditions. Therefore, swiprosin-1 may be a key player in redifferentiation of adult cardiomyocytes.

Next, we investigated the role of calcium in swiprosin-1 dependent spreading. We decreased the intracellular calcium concentration using Bapta-AM. Bapta-AM is a well-known and often used cell-permanent calcium chelator [29]. Binding of free intracellular Ca^2+^ by Bapta-AM led to a decrease of pseudopodia-like structures in ARVC compared to the control group. Furthermore, western blot analysis revealed a reduction of swiprosin-1 on protein level in Bapta-AM treated ARVC. Hence, calcium seems to be required for swiprosin-1 expression and activity.

Finally, we found a strong correlation between swiprosin-1 expression and proteins, participating in β-adrenoceptor desensitization. Swiprosin-1 expression was positively correlated with the expression of GRK5, β1-arrestin, β2-arrestin, and especially GRK2. This relationship between swiprosin-1 and GRK2 was confirmed by Western blot analysis. Its functional relevance was implicated by load free cell shortening. This consequently leads to the assumption that an up-regulation of swiprosin-1, e.g. during cardiac stress, causes a decrease in ARVC responsiveness to β-adrenergic stimulation. β-adrenoceptor desensitization is a characteristic feature of heart failure. Here it is associated with cardiac remodeling and leads to an impaired cardiac function with a depression of heart rate and cardiac output [30]. However, the desensitization of β-adrenoceptors in an acute cardiac insult may have a protective effect [31]. Patients suffering from a myocardial infarction normally receive β-blockers [32–34]. Thus it is expedient for swiprosin-1, which seems to play a major role in the adaptation and regeneration process of ARVC via dedifferentiation and redifferentiation, to be coupled to GRK2 expression, which leads to β-adrenoceptor desensitization. This may protect dedifferentiating cardiomyocytes during the adaptation to stress.

Additionally, we detected a positive correlation of swiprosin-1 with β_1_- and β_2_-adrenoceptors, although the effect was stronger for β_1_. Former studies revealed a shift from β_1_- to β_2_-adrenoceptors during cultivation, which caused an increased hypertrophic responsiveness [17,18].

The role of swiprosin-1 in chronic heart injuries was not part of the experiments shown here and needs to be investigated in the future. However, previous experiments have shown that chronic dedifferentiation in stressed adult cardiomyocytes leads to impaired cardiac function and survival rates. But, in acute states it had positive effects [4,6]. Therefore, we hypothesize that swiprosin-1 and the dedifferentiation process are needed for acute regeneration of adult cardiomyocytes under stress situations. Future investigations will have to focus on the relevance of cardiac swiprosin-1 expression *in vivo*.

## Supporting Information

S1 Fig Categorization of ARVC by light microscopy. Day 0: Freshly isolated cardiomyocytes are „rod-shaped“. Day 2: In the first days of culture ARVC „round down”(1 and 2). Cardiomyocytes with „unusual appearance”(3) showed an irregular surface. Day 6: Cardiomyocytes with a round cell body and pseudopodia-like structures (1) as well as widespread cardiomyocytes (2) were counted as „spreading“. „Unusual appearence”is shown in this picture as (3). Pseudopodia-like structures are shown exemplary.
(TIFF)

S2 Fig DNA gel of qRT-PCR in ARVC. DNA gels showed a mRNA expression of the cardiac specific ANP in ARVC cultivated for five days, whereas an expression of the fibroblast marker collagen 3 could not be detected. Isolated cardiac fibroblasts (FB) served as control. Efhd2 and our house-keeping gene hprt are expressed in both cell types.
(TIFF)

S3 Fig Immunflourescent staining of ARVC treated with siRNA against swiprosin-1. Dedifferentiation and redifferentiation of ARVC treated with siRNA against swiprosin-1. Like controls ARVC lost their contractile apparatus and round down first. This was followed by spreading and reformation of the contractile apparatus with actin-stress fibers and *de novo* sarcomerogenesis.
(TIFF)

S1 Table Sequences and annealing temperatures of all primers used. Shown are all primers used with specific annealing temperature and sequence.
(TIFF)

## References

[1] Li F, Wang X, Capasso JM, Gerdes AM, Rapid transition of cardiac myocytes from hyperplasia to hypertrophy during postnatal development. Journal of molecular and cellular cardiology 1996; 28: 1737–1746. 10.1006/jmcc.1996.0163 8877783

[2] Leri A, Kajstura J, Anversa P, Mechanisms of myocardial regeneration. Trends in cardiovascular medicine 2011; 21: 52–58. 10.1016/j.tcm.2012.02.006 22578241 PMC3356689

[3] Szibor M, Pöling J, Warnecke H, Kubin T, Braun T, Remodeling and dedifferentiation of adult cardiomyocytes during disease and regeneration. Cellular and Molecular Life Sciences 2014; 71: 1907–1916. 10.1007/s00018-013-1535-6 24322910 PMC11113405

[4] Kubin T, Pöling J, Kostin S, Gajawada P, Hein S, Rees W et al, Oncostatin M Is a Major Mediator of Cardiomyocyte Dedifferentiation and Remodeling. Cell Stem Cell 2011; 9: 420–432. 10.1016/j.stem.2011.08.013 22056139

[5] Pöling J, Gajawada P, Lörchner H, Polyakowa V, Szibor M, Böttger T et al, The Janus face of OSM-mediated cardiomyocyte dedifferentiation during cardiac repair and disease. Cell Cycle 2014; 11: 439–445. 10.4161/cc.11.3.19024 22262173

[6] Pöling J, Gajawada P, Richter M, Lörchner H, Polyakova V, Kostin S et al, Therapeutic targeting of the oncostatin M receptor-β prevents inflammatory heart failure. Basic Res Cardiol 2014; 109: 1–14. 10.1007/s00395-013-0396-3 24292852

[7] Schwarzfeld T, Isolation and development in cell culture of myocardial cells of the adult rat. Journal of molecular and cellular cardiology 1981; 13: 563–575. 7277505 10.1016/0022-2828(81)90327-8

[8] Eppenberger-Eberhardt M, Flamme I, Kurer V, Eppenberger HM, Reexpression of α-smooth muscle actin isoform in cultured adult rat cardiomyocytes. Developmental Biology 1990; 139: 269–278. 2186943 10.1016/0012-1606(90)90296-u

[9] Eppenberger ME, Hauser I, Baechi T, Schaub MC, Brunner UT, Dechesne CA et al, Immunocytochemical analysis of the regeneration of myofibrils in long-term cultures of adult cardiomyocytes of the rat. Developmental Biology 1988; 130: 1–15. 2903104 10.1016/0012-1606(88)90408-3

[10] Huh YH, Kim SH, Chung KH, Oh S, Kwon MS, Choi HW et al, Swiprosin-1 modulates actin dynamics by regulating the F-actin accessibility to cofilin. Cellular and Molecular Life Sciences 2013; 70: 4841–4854. 10.1007/s00018-013-1447-5 23959172 PMC3830201

[11] Dütting S, Brachs S, Mielenz D, Fraternal twins: Swiprosin-1/EFhd2 and Swiprosin-2/EFhd1, two homologous EF-hand containing calcium binding adaptor proteins with distinct functions. Cell communication and signaling 2011; 9: 2 10.1186/1478-811X-9-2 21244694 PMC3036668

[12] Kwon MS, Park KR, Kim YD, Na BR, Kim HR, Choi HJ et al, Swiprosin-1 is a novel actin bundling protein that regulates cell spreading and migration. PLoS ONE 2013; 8 e71626 10.1371/journal.pone.0071626 23977092 PMC3744483

[13] Fiedler B, Wollert KC, Interference of antihypertrophic molecules and signaling pathways with the Ca2+-calcineurin-NFAT cascade in cardiac myocytes. Cardiovascular Research 2004; 63: 450–457. 10.1016/j.cardiores.2004.04.002 15276470

[14] Fiedler B, Wollert KC, Targeting calcineurin and associated pathways in cardiac hypertrophy and failure. Expert opinion on therapeutic targets 2005; 9: 963–973. 10.1517/14728222.9.5.963 16185152

[15] Lim HW, Molkentin JD, Calcineurin and human heart failure. Nature medicine 1999; 5: 246–247. 10.1038/6430 10086361

[16] Heineke J, Ruetten H, Willenbockel C, Gross SC, Naguib M, Schaefer A et al, Attenuation of cardiac remodeling after myocardial infarction by muscle LIM protein-calcineurin signaling at the sarcomeric Z-disc. Proceedings of the National Academy of Sciences of the United States of America 2005; 102: 1655–1660. 10.1073/pnas.0405488102 15665106 PMC547821

[17] Schluter KD, Zhou XJ, Piper HM, Induction of hypertrophic responsiveness to isoproterenol by TGF-beta in adult rat cardiomyocytes. The American journal of physiology 1995; 269: 6. 10.1152/ajpcell.1995.269.5.C1311 7491923

[18] Zhou XJ, Schluter KD, Piper HM, Hypertrophic responsiveness to beta 2-adrenoceptor stimulation on adult ventricular cardiomyocytes. Molecular and cellular biochemistry 1996; 163–164: 211–216. 8974059 10.1007/BF00408660

[19] Joshi-Mukherjee R, Dick IE, Liu T, O'Rourke B, Yue DT, Tung L et al, Structural and functional plasticity in long-term cultures of adult ventricular myocytes. Journal of molecular and cellular cardiology 2013; 65: 76–87. 10.1016/j.yjmcc.2013.09.009 24076394 PMC4219275

[20] Schlüter KD, Schreiber D, Adult Ventricular Cardiomyocytes: Isolation and Culture, in: Fabbro D., McCormick F. (Eds.), Protein Tyrosine Kinases: From Inhibitors to Useful Drugs. Humana Press Inc, Totowa, NJ, 2005; 305–314. 10.1385/1-59259-838-2:305 15361670

[21] Schluter KD, Katzer C, Frischkopf K, Wenzel S, Taimor G, Piper HM, Expression, release, and biological activity of parathyroid hormone-related peptide from coronary endothelial cells. Circulation Research 2000; 86: 946–951. 10807866 10.1161/01.res.86.9.946

[22] Anwar A, Schluter KD, Heger J, Piper HM, Euler G, Enhanced SERCA2A expression improves contractile performance of ventricular cardiomyocytes of rat under adrenergic stimulation. Pflugers Archiv European journal of physiology 2008; 457: 485–491. 10.1007/s00424-008-0520-7 18581135

[23] Schreckenberg R, Dyukova E, Sitdikova G, Abdallah Y, Schluter KD, Mechanisms by which calcium receptor stimulation modifies electromechanical coupling in isolated ventricular cardiomyocytes. Pflugers Archiv European journal of physiology 2015; 467: 379–388. 10.1007/s00424-014-1498-y 24687204

[24] Fontana M, Olschewski H, Olschewski A, Schluter KD, Treprostinil potentiates the positive inotropic effect of catecholamines in adult rat ventricular cardiomyocytes. British journal of pharmacology 2007; 151: 779–786. 10.1038/sj.bjp.0707300 17533419 PMC2014129

[25] Bugaisky LB, Zak R, Differentiation of Adult Rat Cardiac Myocytes in Cell Culture, Circulation Research 1989; 64: 493–500. 2465096 10.1161/01.res.64.3.493

[26] Decker ML, Behnke-Barclay M, Cook MG, Lesch M, Decker RS, Morphometric evaluation of the contractile apparatus in primary cultures of rabbit cardiac myocytes. Circulation Research 1991; 69: 86–94. 2054944 10.1161/01.res.69.1.86

[27] Schlüter KD (Ed.), Cardiomyocytes–Active Players in Cardiac Disease, Springer International Publishing AG Switzerland, 2016, ISBN 978-3-319-31249-1

[28] Hilenski LL, Terracio L, Borg TK, Myofibrillar and cytoskeletal assembly in neonatal rat cardiac myocytes cultured on laminin and collagen. Cell Tissue Research 1991; 264: 577–587. 1907887 10.1007/BF00319047

[29] Tsien RS, A non-disruptive technique for loading calcium buffers and indicators into cells. Nature 1981; 290: 527–528. 7219539 10.1038/290527a0

[30] Mason RP, Giles TD, Sowers JR, Evolving mechanisms of action of beta blockers: focus on nebivolol. Journal of cardiovascular pharmacology 2009; 54: 123–128. 10.1097/FJC.0b013e3181ad207b 19528811

[31] Najafi A, Sequeira V, Kuster DW, van der Velden J, beta-adrenergic receptor signalling and its functional consequences in the diseased heart. European journal of clinical investigation 2016; 46: 362–374. 10.1111/eci.12598 26842371

[32] Howard PA, Ellerbeck EF, Optimizing beta-blocker use after myocardial infarction. American family physician 2000; 62: 1853–60, 1865–6. 11057841

[33] Kezerashvili A, Marzo K, de Leon J, Beta blocker use after acute myocardial infarction in the patient with normal systolic function: when is it "ok" to discontinue? Current cardiology reviews 2012; 8: 77–84. 10.2174/157340312801215764 22845818 PMC3394111

[34] Lange R, Kloner RA, Braunwald E, First ultra-short-acting beta-adrenergic blocking agent: its effect on size and segmental wall dynamics of reperfused myocardial infarcts in dogs. The American journal of cardiology 1983; 51: 1759–1767. 6134464 10.1016/0002-9149(83)90224-2

