# Supplementary figures and images for: The Effects of Swiprosin-1 on the Formation of Pseudopodia-Like Structures and β-Adrenoceptor Coupling in Cultured Adult Rat Ventricular Cardiomyocytes
Source: PLoS One. 2016 Dec 16;11(12):e0167655. doi: 10.1371/journal.pone.0167655 (PMC5161327; doi:10.1371/journal.pone.0167655)

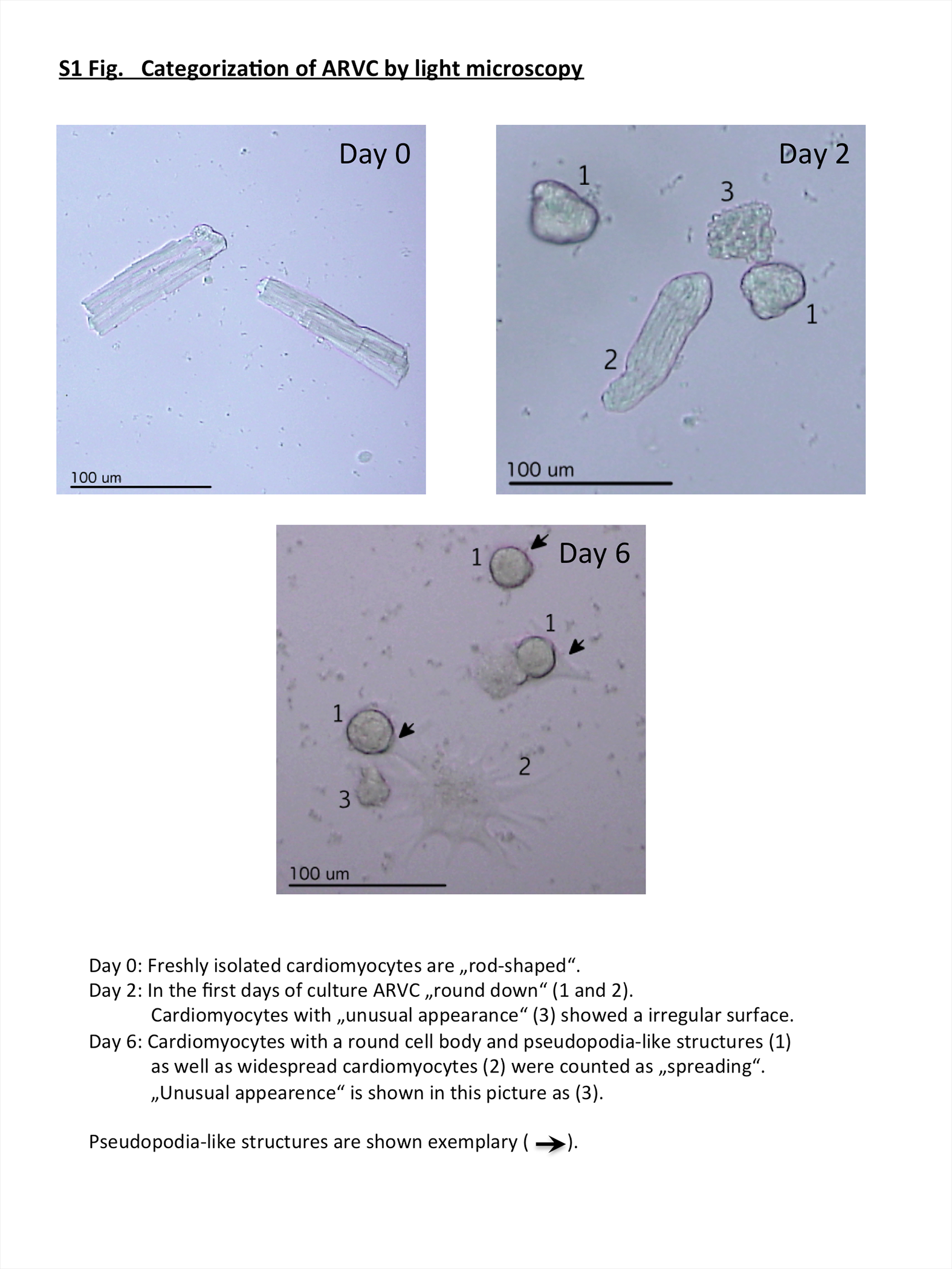

Supplement: S1 Fig — Day 0: Freshly isolated cardiomyocytes are „rod-shaped“. Day 2: In the first days of culture ARVC „round down”(1 and 2). Cardiomyocytes with „unusual appearance”(3) showed an irregular surface. Day 6: Cardiomyocytes with a round cell body and pseudopodia-like structures (1) as well as widespread cardiomyocytes (2) were counted as „spreading“. „Unusual appearence”is shown in this picture as (3). Pseudopodia-like structures are shown exemplary. (TIFF) [file pone.0167655.s001.tiff]

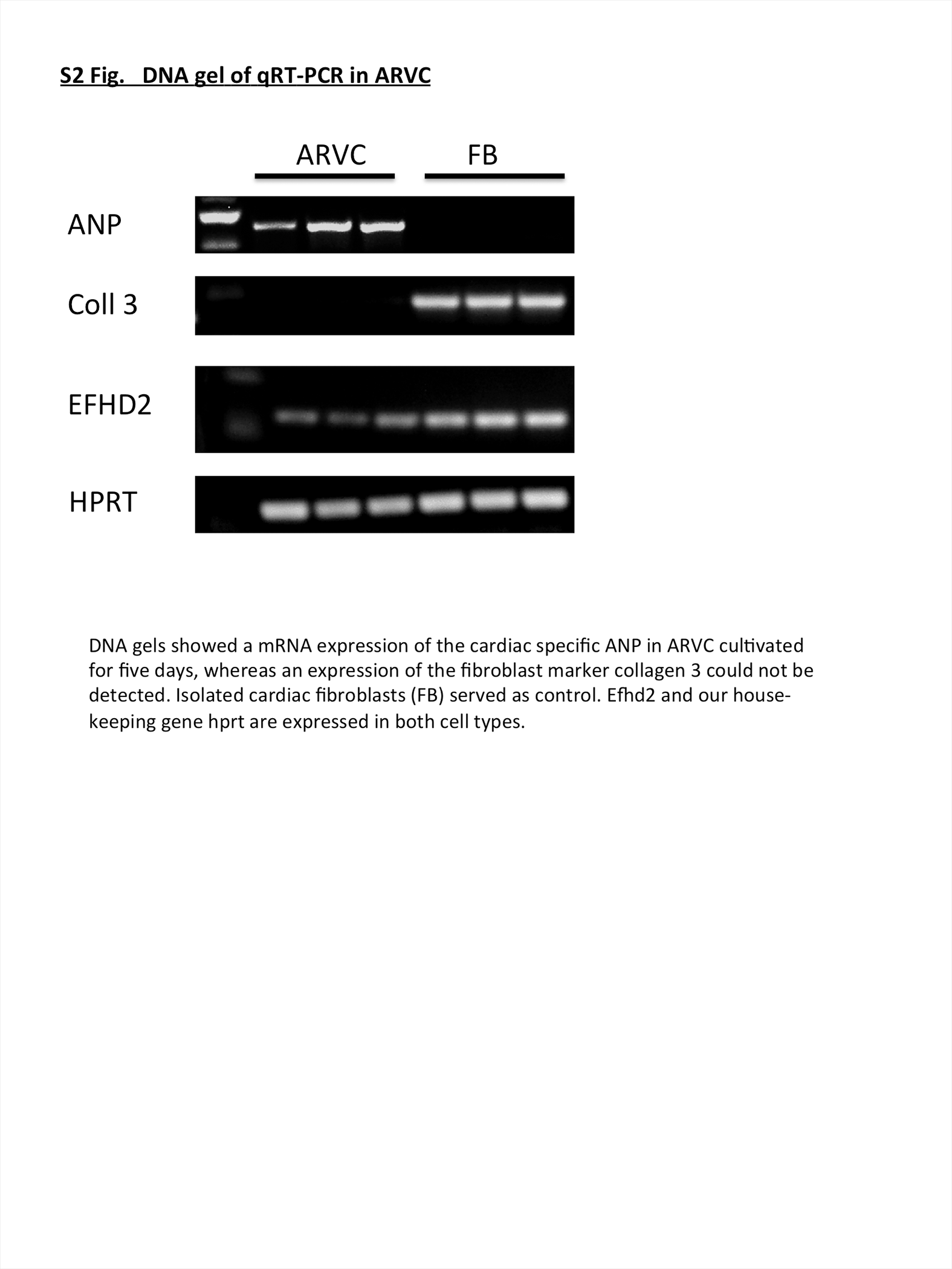

Supplement: S2 Fig — DNA gels showed a mRNA expression of the cardiac specific ANP in ARVC cultivated for five days, whereas an expression of the fibroblast marker collagen 3 could not be detected. Isolated cardiac fibroblasts (FB) served as control. Efhd2 and our house-keeping gene hprt are expressed in both cell types. (TIFF) [file pone.0167655.s002.tiff]

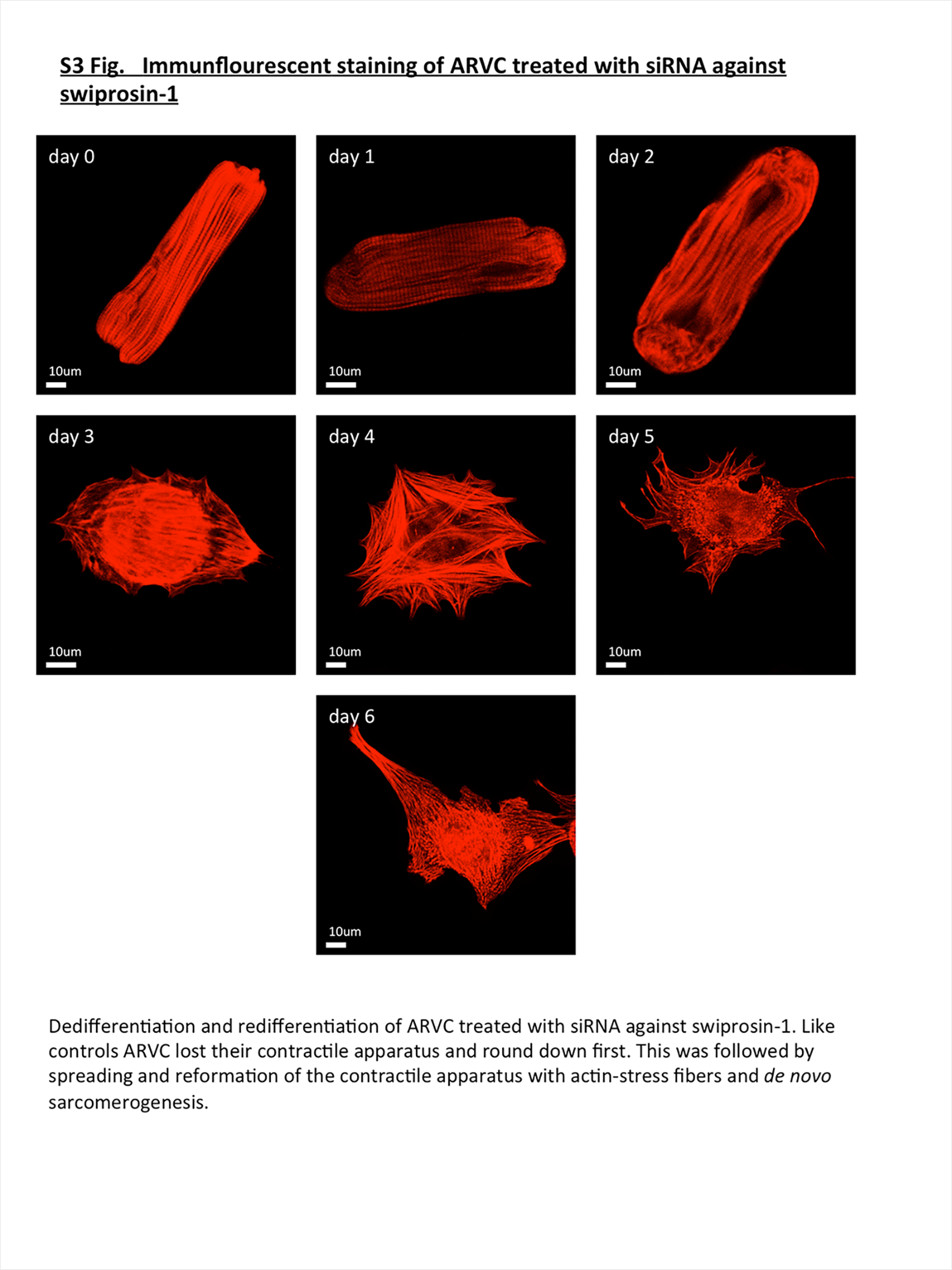

Supplement: S3 Fig — Dedifferentiation and redifferentiation of ARVC treated with siRNA against swiprosin-1. Like controls ARVC lost their contractile apparatus and round down first. This was followed by spreading and reformation of the contractile apparatus with actin-stress fibers and de novo sarcomerogenesis. (TIFF) [file pone.0167655.s003.tiff]

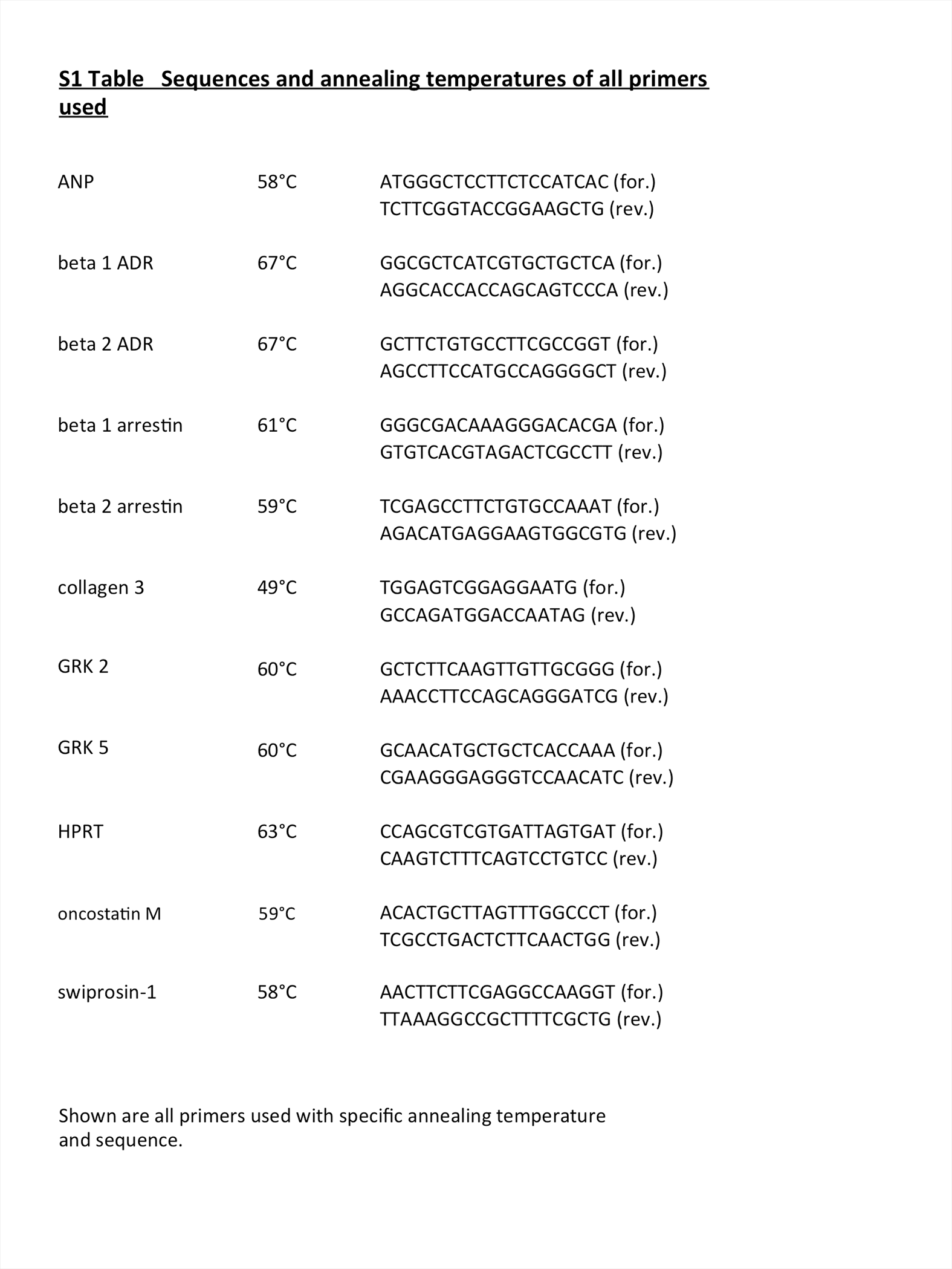

Supplement: S1 Table — Shown are all primers used with specific annealing temperature and sequence. (TIFF) [file pone.0167655.s004.tiff]
